# Intercomparison of *in-situ* aircraft and satellite aerosol measurements in the stratosphere

**DOI:** 10.1038/s41598-019-52089-6

**Published:** 2019-10-30

**Authors:** Oscar S. Sandvik, Johan Friberg, Bengt G. Martinsson, Peter F. J. van Velthoven, Markus Hermann, Andreas Zahn

**Affiliations:** 1 Division of Nuclear Physics, Lund University, Lund, Sweden; 2 Royal Netherlands Meteorological Institute (KNMI), De Bilt, the Netherlands; 3 Leibniz Institute for Tropospheric Research, Leipzig, Germany; 4 Institute of Meteorology and Climate Research, Institute of Technology, Karlsruhe, Germany

**Keywords:** Atmospheric chemistry, Atmospheric dynamics, Atmospheric chemistry

## Abstract

Aerosol composition and optical scattering from particles in the lowermost stratosphere (LMS) have been studied by comparing *in-situ* aerosol samples from the IAGOS-CARIBIC passenger aircraft with vertical profiles of aerosol backscattering obtained from the CALIOP lidar aboard the CALIPSO satellite. Concentrations of the dominating fractions of the stratospheric aerosol, being sulphur and carbon, have been obtained from post-flight analysis of IAGOS-CARIBIC aerosol samples. This information together with literature data on black carbon concentrations were used to calculate the aerosol backscattering which subsequently is compared with measurements by CALIOP. Vertical optical profiles were taken in an altitude range of several kilometres from and above the northern hemispheric extratropical tropopause for the years 2006-2014. We find that the two vastly different measurement platforms yield different aerosol backscattering, especially close to the tropopause where the influence from tropospheric aerosol is strong. The best agreement is found when the LMS is affected by volcanism, i.e., at elevated aerosol loadings. At background conditions, best agreement is obtained some distance (>2 km) above the tropopause in winter and spring, i.e., at likewise elevated aerosol loadings from subsiding aerosol-rich stratospheric air. This is to our knowledge the first time the CALIPSO lidar measurements have been compared to *in-situ* long-term aerosol measurements.

## Introduction

Aerosols from volcanic eruptions can significantly affect the Earth’s radiation budget and thus the Earth’s climate^1–4^ due to aerosol scattering of incoming solar radiation. A well-known historic example of a major volcanic eruption with this cooling effect is the ‘year without a summer’ in 1816 when the 1815 Tambora volcanic eruption spread aerosols across the globe^5–7^. A more recent example with global impact is the 1991 Mt. Pinatubo eruption, leading to a decrease in the tropospheric temperature of more than 0.7 °C in the year following the eruption^1^.

Even medium-sized volcanic eruptions (having a Volcanic Explosivity Index (VEI) ≤ 4) can cause significant impacts and thus should be taken into account for realistic climate modelling^8,9^. During the first decade of the 21^st^ century, the aerosol from several medium-sized volcanic eruptions is believed to have contributed to a slowdown in global warming causing most global climate models to overestimate the rise in global mean surface temperatures during this period^8^. Importantly, these climate models have not considered the aerosol in the lowermost stratosphere (LMS). Indeed, it has only recently been shown that a significant fraction of the stratospheric aerosol optical depth (AOD) is located in the LMS^10–12^. In both major and medium-sized eruptions, the most important aerosol related emissions from volcanoes are ash and sulphur dioxide (SO_2_). Volcanic ash usually separates from gaseous sulphur dioxide and sediments in short time, whereas sulphur dioxide, in particular when penetrating the tropopause, can remain in the atmosphere for significantly longer times^13^ and thus can be distributed globally in the stratosphere. Sulphur dioxide emitted from major volcanic eruptions form sulphate aerosol^2,14^. Volcanic aerosols primarily consist of sulphuric acid/water particles^15^, with some additional ash and organic material^16–19^.

The tropopause is located at higher altitudes in the tropics than in the extra-tropics^20^. The potential temperature at the tropical tropopause is around 380 K and at higher latitudes this isentrope is in the stratosphere, well above the tropopause. The LMS is specifically defined as the air mass between the tropopause and the 380 K isentrope^21^. Upper stratospheric air enters the LMS from above, whereas tropospheric air enters the LMS from below through local vertical mixing and sideways through isentropic mixing, mainly in the subtropics^20^. Trajectory analysis by Haynes and Shuckburgh^22^ and Berthet, *et al*.^23^ has shown that near the subtropical jet-stream there is a latitudinal barrier for such isentropic air transport from the tropics northwards into the LMS. This barrier is weakened during summer. The LMS contains a larger fraction of stratospheric air during winter and spring than during summer and autumn^20,24^.

Sulphur can enter the stratosphere through other ways than direct injections by volcanic eruptions. The dominant pathway is the transport of sulphur-containing gases, primarily carbonyl sulphide (OCS) and some sulphur dioxide (SO_2_), into the tropical stratosphere and the subsequent dispersion within the Brewer-Dobson (BD) circulation where they are finally oxidized to H_2_SO_4_^25^. The stratospheric background aerosol, the so-called Junge layer^26,27^, stems from gas-to-particle conversion of the oxidation products of OCS and other sulphur-containing species in the middle stratosphere^27^. Stratospheric air is thus rich in sulphuric aerosol particle mass compared to the upper tropospheric air. This leads to a concentration gradient of sulphuric aerosol in the LMS which is modulated by seasonal variation of the BD circulation and the exchange of air across the tropopause^28^. The stratospheric aerosol also contains an organic fraction and a small black carbon fraction, which affect the optical properties of the particles^29,30^. Sulphurous aerosol in the tropopause region also shows this seasonal variation because during the first six months of a year the down-transported stratospheric air dominates and during the remaining six months the tropospheric air dominates^31^.

Measurements of aerosol particles in the stratosphere have been performed for several decades either *in-situ* using balloons^26,32^ or continuously from afar by satellite and ground-based remote sensing^27,33^. Stratospheric aerosol, measured between 2006 and 2014, is the focus of this article, where vertical profiles of aerosol backscattering in the LMS are investigated. To this end, two unique complimentary observational systems have been used: (a) aerosol samples collected by the “In-service Aircraft for a Global Observing System – Civil Aircraft for the Regular Investigation of the atmosphere Based on an Instrument Container” (IAGOS-CARIBIC; https://www.iagos.org/iagos-caribic/, a European Research Infrastructure)^34^, and (b) the Cloud-Aerosol Lidar with Orthogonal Polarization (CALIOP) aboard the Cloud-Aerosol Lidar and Infrared Pathfinder Satellite Observation (CALIPSO)^35^. Both observation systems provide long-term aerosol measurements with high vertical resolution and good coverage in the northern hemispheric LMS. This combination is unmatched by other satellites and *in-situ* samplers. We compared aerosol backscatter signals from CALIOP with those derived using the aerosol particle elemental mass concentrations obtained from IAGOS-CARIBIC with respect to troposphere-stratosphere exchange and influence from volcanic aerosol.

## Methods

### IAGOS-CARIBIC

#### *In-situ* sampling of aerosols during commercial flights

The IAGOS-CARIBIC observatory^34^ aboard an in-service Lufthansa Airbus A340-600 was used to collect aerosol samples by impaction during intercontinental flights. Many trace gases and other parameters are measured *in situ* in parallel, such as water vapour, ozone and the submicrometer particle size distribution^36–39^. In this paper we have primarily analysed the aerosol samples, but we also used the ozone and H_2_O measurements^34,40^ and the particle size data from the optical particle size spectrometer OPSS). The IAGOS-CARIBIC observatory (a modified air freight container) is loaded once per month into the cargo bay of the aircraft for measurements during four consecutive inter-continental flights. Air for aerosol sampling is led through a dedicated tip-heated aerosol inlet system to a cyclone. The cyclone removes particles having an aerodynamic diameter larger than 2 μm (50% cut-off), and the remaining particles are led to a multi-channel impactor system^41^. The multi-channel system provides several consecutive samples each flight, with each sample having a sampling time of typically 100 min. The lower threshold aerodynamic diameter is 0.08 μm and the collection efficiency is close to 100%^41^. The samples have previously been compared to the IAGOS-CARIBIC OPSS with good agreement, where the ratios between particle volume from the OPSS and the total particulate mass derived from the impactor samples are within a narrow interval for 84% of the samples^42^. Samples were taken between 9.5 km and 12 km altitude during 95% of the flights used here (https://www.iagos.org/iagos-caribic/ (2019)), and this altitude interval was also used for analysing the CALIOP data. For logistical reasons, most IAGOS-CARIBIC flights are in the northern hemisphere where at passenger aircraft flight altitudes the aircraft frequently enters the LMS. The present analysis has been restricted to 30 − 70°N where we have observations in the LMS by both methods.

#### PIXE and PESA analysis

Determination of elemental concentrations in the IAGOS-CARIBIC aerosol samples was conducted using particle-induced X-ray emission (PIXE) and particle elastic scattering analysis (PESA)^42^. These two accelerator based analysis methods, performed at the Lund Ion Beam Analysis Facility in Sweden using a proton beam of 2.55 MeV, yield low minimum detection limits which are suitable for upper tropospheric and stratospheric aerosol samples with low particle mass concentrations.

In this study we have used the particulate sulphur and carbon concentrations (given in ng m^−3^ at standard (STP) conditions of 273.15 K and 1013 hPa) obtained from PIXE/PESA analysis to estimate the aerosol scattering at the altitudes where the samples were taken. The methodology is described in the next section. The minimum detection limits for sulphur and carbon are 2 and 15 ng m^−3^ STP^29^, respectively. The combined uncertainty from sampling and analysis is estimated to be 12%^42^. Further details about sampling and analyses can be found in Nguyen, *et al*.^41^ and Martinsson, *et al*.^42^.

#### Particle composition, size distribution, hygroscopic growth and optical properties

Although the temperatures in the LMS usually are well below that of homogeneous freezing of water, the sulphuric acid particles are highly concentrated liquids because of the dry conditions^43^. During IAGOS-CARIBIC flights, two water sensors are in use since 2006^36^; one chilled mirror frost point hygrometer (FPH) for gas phase H_2_O and a two-channel photoacoustic laser spectrometer (PAS) for gas phase H_2_O and total H_2_O with a time resolution of 5 s. The PAS data are calibrated post flights using the data from the FPH, having an uncertainty of 0.5 K. The FPH is regularly checked against a high precision FPH instrument (MWB LX-373) in the lab. The uncertainty of the H_2_O measurements is the highest of 4% and 0.3 ppmv^36^. Based on the H_2_O measurements in the gas phase and measured temperature, the relative humidity (RH) was obtained assuming a super-cooled liquid^44^. By matching the water activity to the relative humidity, the molality of the H_2_SO_4_/H_2_O aerosol, and hence weight fraction of H_2_SO_4_, was obtained from a parameterization^45^ of model results by Clegg and Brimblecombe^46^. The density (*ρ*_*a*_) of the H_2_SO_4_/H_2_O solution was obtained using data in Myhre, *et al*.^47^ Here we made a simplified parameterization

$${\rho }_{a}({T}_{a})=({a}_{0}\,+\,{a}_{1}{w}_{a}){T}_{a}\,+\,{b}_{0}\,+\,{b}_{1}{w}_{a}\,+\,{b}_{2}{w}_{a}^{2}$$

where *T*_*a*_ is the temperature in the atmosphere and *w*_*a*_ the mass fraction of H_2_SO_4_ at atmospheric conditions, and the constants (*a*_0_, *a*_1_, *b*_0_, *b*_1_, *b*_2_) with values of (−0.4845, −0.7074, 1186.1, 621.4, 573.54) in kg m^−3^ K^−1^ (*a*_*i*_) and kg m^−3^ (*b*_*i*_). The parameterization reproduces the density data by 0.6% or better. From the weight fractions of sulphuric acid and its density, the atmospheric volume concentration of H_2_SO_4_/H_2_O aerosol is obtained.

The stratospheric aerosol is dominated by sulphuric acid and water. Still, the LMS aerosol also contains a considerable fraction of carbonaceous aerosol^16,48^ that is mainly organic^29^, which, at least in part, is mixed with the sulphurous aerosol^49^. The physical and chemical properties of the carbonaceous fraction of the LMS aerosol is not well known. Still, we had to add an organic fraction to the H_2_SO_4_/H_2_O aerosol volume, based on the carbon concentration. Relying on the stoichiometric relations between sulphur, oxygen and carbon^29^ the carbon concentration in each sample was multiplied by 1.25 to obtain an estimate of the concentration of organic aerosol, with the density assumed to be 1200 kg m^−3^ ^50–52^. These values will be further investigated in an uncertainty analysis. The stratospheric aerosol also contains a small fraction of black carbon (BC) with the estimated concentration of 1 ng BC per kg of air, based on literature data^53–56^. The density is estimated to 1800 kg m^−3^ ^57^. The BC concentration was subtracted from the measured carbon concentration prior to the estimate of the organic aerosol concentration.

Particle size distributions are measured in IAGOS-CARIBIC using an OPSS in the diameter range 0.1–1 µm^39^. Data are available starting from year 2010, implying that data are missing from approximately half of time period studied here. Therefore, we cannot use individual particle size data for each measurement. Instead we use the average distribution for one year (volume geometrical mean diameter: *D*_*g,v*_ = 321 nm, and geometrical standard deviation: *σ*_*g*_ 1.52) and its variability. The period (April 2011 to March 2012) included close to background conditions as well as two intermediate volcanic eruptions (Grimsvötn, May 2011 and Nabro, June 2011), inducing minor variation in the size distribution^42^. The size distribution measurements were undertaken after the aerosol passed a tip-heated inlet and the sampling line, with the OPSS at typically 29 °C, and the OPSS operation pressure typically 40% higher than the ambient atmospheric pressure due to the dynamic pressure increase in the inlet system. Measured temperature and pressure in the sampling line are available for all samples. Because of the changed conditions in the OPSS, the air is significantly dryer there than in the atmosphere, resulting in a shrinkage of the particles due to loss of water^39^. For calculation of the particle water content in these dry conditions in the OPSS, other parameterizations than those used for atmospheric conditions are needed for the mass fraction sulphuric acid in the H_2_SO_4_/H_2_O part of the aerosol^58^ and its density^59^.

The refractive index (RI) of the particles was computed based on volume mixing of the constituents. The RI of the H_2_SO_4_/H_2_O component was based on data and methodology of Steele and Hamill^60^. For the organic fraction we used the refractive index 1.55 – 0i^61–63^, and for BC 1.95 – 0.79i^30,57^. Each sample has its RI based on the composition. From that RI the particle size-dependent backscattering efficiency is obtained from a Mie scattering code based on Bohren and Huffman^64^ (address: https://www.igf.fuw.edu.pl/%7ekmark/stacja/kody.php, retrieved: 2019-04-24). The computed backscattering based on IAGOS-CARIBIC elemental concentration measurements and the computations described in this section are then compared with measurements from CALIOP.

### CALIOP

#### Satellite-based lidar

The nadir-viewing CALIOP lidar carried aboard the CALIPSO satellite, launched in 2006, provides vertical profiles of backscatter measurements with coverage between 82°N and 82°S^65^. The satellite follows a sun-synchronous orbit with a repeat cycle of 16 days. The vertical resolution of CALIOP can be as low as 30 m at low altitudes but in this article the data were averaged to a resolution of 180 m to get a better signal to noise ratio. The lidar system for the 532 nm wavelength is polarisation sensitive and thus provides information about the shape of particles. Aerosol backscattering was calculated from backscattering data in the night-time, which has better signal-to-noise ratios than the day-time data.

In this study, we used the latest data version of the level 1B CALIOP data, version 4.10. Data versions prior to the 4.00 underestimated the optical extinctions due to a data calibration which was erroneously done at particle-containing altitudes^66^, and contained biases which have been corrected for in version 4.00 and later^67^. Comparison of this improved version with collocated measurements by the airborne High Spectral Resolution Lidar (HSRL) shows that the CALIOP measurements have a relative bias of 1.6% ± 2.4% compared to HSRL^67^. A modern lidar setup has a typical uncertainty of 5-10% for the particulate backscatter coefficient^68^.

#### Cloud filtering

The volume depolarisation ratio was used to filter out clouds and also to distinguish aerosol types. A low depolarisation ratio indicates particles with a spherical shape, such as stratospheric sulphate aerosol, and a large depolarisation ratio indicates particles with non-spherical shapes, such as ice and dust particles^69,70^. To minimize the influence of clouds on the signal we created a cloud mask similar to that of Vernier, *et al*.^66^ using a depolarisation threshold of 5%. The cloud mask was produced in a vertical resolution of 60 m. In order for a cell to be classified as a cloud pixel it needs to have more total backscattering than 2.5 × 10^−4^ km^−1^ sr^−1^ as well as to exceed the depolarisation threshold. The cloud mask was expanded around clouds to capture diffuse cloud edges. All cells beneath groups of cloud cells were removed in order to avoid using cells probed with a severely attenuated beam. The cloud filtering rejects 12.7% of the data used for the present work.

#### CALIOP data processing

The processing of CALIOP data is based on the methods described in Andersson, *et al*.^10^ and Friberg, *et al*.^11^ Horizontal averaging was done along each swath, casting the data into 1° latitudinal averages. Also, the vertical dimension was transformed from altitude above sea level to altitude above the tropopause using potential vorticity data described in the next section. Data cells containing clouds, or were located below the tropopause, or were outside the IAGOS-CARIBIC flight altitudes were excluded in the present work.

Since conditions in the atmosphere and the altitude of the tropopause varies over time, the temperature and pressure for a cell at a specific distance above the tropopause also varies. To compare data from different swaths it is necessary to transfer the data to STP conditions. Therefore, the aerosol backscattering data were transferred to STP (defined as 273.15 K, 1013 hPa) conditions using MERRA-2^71^ temperature and pressure data provided in the CALIOP data. Latitudinal (30–70°N) averages over individual swaths of aerosol backscattering were then calculated with data in latitudes closer to the equator given more weight than the more poleward latitudes since the more equatorial latitudes cover more distance. Then monthly averages were calculated over the latitudinal averages. The aerosol backscattering was then calculated at the actual atmospheric average temperature and pressure conditions. By averaging the data over wide horizontal and vertical distances and over time, the CALIOP minimum detection limit decreases^72^.

### Air-mass classification

To separate air masses between the troposphere and stratosphere the dynamical tropopause^20^ has been used throughout this article. It is based on the potential vorticity (PV; unit 1 PVU = 10^−6^ K m^2^ kg^−1^ s^−1^), a parameter that describes the dynamic stability of air masses. The threshold value for potential vorticity defining the tropopause is often set to 2 PVU^20^. In this study, the threshold was set to 1.5 PVU in order to observe the mixing between stratospheric and tropospheric air and ensure that characteristic stratospheric air remains above the threshold. To get PV values for each cell in the satellite swaths, the ECMWF ERA Interim product was interpolated into the CALIOP grid. The interpolated PV values were then used to identify the cell containing the tropopause in the CALIOP data. For the IAGOS-CARIBIC measurements, the altitude above the tropopause was obtained from the altitude of the aircraft and the altitude of the tropopause from the ECMWF ERA interim^31^.

Volcanic aerosol is readily observed by both CALIOP and IAGOS-CARIBIC. The long lifetime of the volcanic aerosol in the stratosphere results in clearly higher aerosol backscattering in the months following major volcanic eruptions than in the months with background conditions. In this study, each month has been labelled as either more or less volcanically affected depending on the elapsed time since a major eruption.

## Results

### Hygroscopic growth and particle size

Hygroscopic growth, particle size distribution, and particle composition are important parameters determining the aerosol backscattering measured by CALIOP. Here we present results on relative humidity over a liquid solution (see the Methods section), water uptake by sulphuric acid and effects on particle size due to the different conditions in the atmosphere compared with the conditions at the OPSS for particle size distribution measurements.

Table 1 shows the average conditions in the atmosphere and OPSS for three layers in the LMS. As we go deeper into the LMS, the pressure (*p*_*a*_) decreases and the temperature (*T*_*a*_) increases as expected. The relative humidity for liquid solution (RH_*a*_) decreases with depth into the LMS, manifesting the increasing influence from dry stratospheric air. This, in turn, leads to decreasing water activity in the particles, and thus increasing fractions of sulfuric acid (*w*_*a*_) in the H_2_SO_4_/H_2_O part of the particles.

**Table 1:** Average conditions in the atmosphere (a) and during sizing of particles in the IAGOS-CARIBIC optical particle size spectrometer (OPSS).

Altitude above the tropopause (m)	300–1000	1000–2000	>2000
*Conditions in the OPSS:*			
*p*_OPSS_ (hPa)^a^	331	326	315
*T*_OPSS_ (K)^b^	302	302	303
RH_OPSS_ (%)^c^	0.039	0.023	0.009
*w*_OPSS_ (%)^d^	88	90	93
*Conditions in the atmosphere:*			
*p*_*a*_ (hPa)^a^	236	232	226
*T*_*a*_ (K)^b^	219	220	223
RH_*a*_ (%)^c^	27	14	4
*w*_*a*_ (%)^d^	47	55	65
*D*_*g,v,a*_/*D*_*g,v*,OPSS_^e^	1.23	1.18	1.12

^a^Pressure, ^b^temperature, ^c^relative humidity, ^d^weight percent of H_2_SO_4_ in the H_2_SO_4_/H_2_O part of the particles, ^e^volume geometrical mean diameter.

For the OPSS measurements, the slight compression in the measurement line (*p*_OPSS_) due to the dynamical pressure acting on the inlet, which increases the water volume concentration, has a much smaller influence on the relative humidity (RH_OPSS_) than the increase in the saturation vapour pressure due to the strong elevation in temperature (*T*_OPSS_). As a result, the conditions in the OPSS become very dry. This in turn, leads to evaporation of water^39^, and the fractions of sulphuric acid (*w*_OPSS_) in the H_2_SO_4_/H_2_O part of the particles become very large. The volume of the H_2_SO_4_/H_2_O part of the particles are on average factors 2.3 (LMS layer 300–1000 m above the tropopause), 1.9 (1000–2000 m) and 1.6 (>2000 m) larger in the atmosphere than in the OPSS, corresponding to factors 1.33, 1.25 and 1.17 in terms of diameter, respectively. This volume change does not fully affect the particle size because of the presence of other constituents, mainly organics, which are assumed not to affect the water uptake (see the Methods section). The particle size measured by the OPSS are on average estimated to be a factor of 1.23 to 1.12 smaller, depending on LMS layer, than the size in the atmosphere (Table 1).

The above computations combined with assumptions and calculations of optical properties are described in the Methods section, and tested as described in a section below. They form the basis for modelling the backscattering of the aerosol in the LMS from aerosol samples and other measurements undertaken by IAGOS-CARIBIC. In the following sections this modelled backscattering will be compared with measured backscattering from the satellite-based lidar CALIOP.

### General tendencies

**Figure 1:**
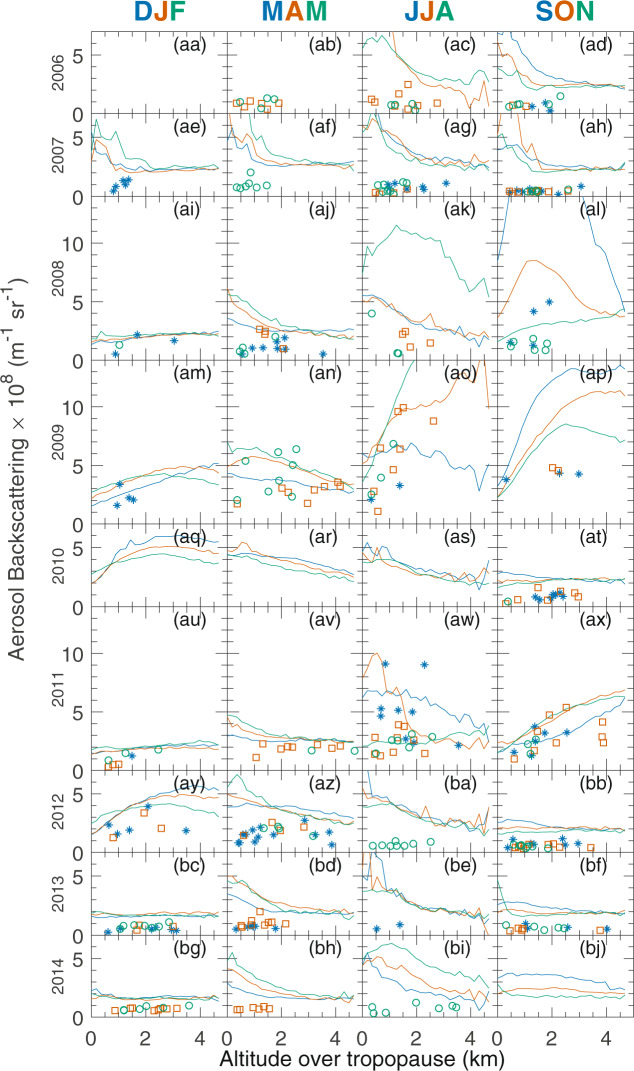
Aerosol backscattering versus altitude above the tropopause calculated from aerosol samples (IAGOS-CARIBIC) and directly measured by lidar (CALIOP). The solid lines are the monthly mean values for CALIOP and each dot is an individual sample from IAGOS-CARIBIC. Blue, vermillion and green lines and marks indicate data for the first, second and third months of a season, respectively. The data were taken between 30°N and 70°N. The CALIOP data were averaged over all longitudes and in the same latitude and altitude (9.5 to 12 km) intervals as IAGOS-CARIBIC. CALIOP data have been cloud filtered.

Vertical profiles of aerosol backscattering from CALIOP satellite lidar and *in-situ* IAGOS-CARIBIC measurements are plotted in Fig. 1. Each subfigure represents a season with three colour coded-months showing the aerosol backscattering against altitude above the tropopause. All IAGOS-CARIBIC measurements in a month are expressed as dots and the monthly averages from CALIOP are expressed as lines. It is important to realize that point measurements from IAGOS-CARIBIC, with patchiness due to variable concentrations in the atmosphere, are compared with averages from CALIOP spanning entire months and a large area of the Earth (30–70°N). A single IAGOS-CARIBIC measurement can thus not be compared with averages from CALIOP measurements. Instead, this study relies on the use of a large number of point measurements to obtain statistically relevant comparisons. This figure comprises the basis of the results in this article and most subsequent analyses will be made from this figure. The CALIOP nadir angle was 0.3° before November 2007. With this nadir angle, the platform experienced increased uncertainties due to specular reflection from horizontally oriented ice crystals^65^. Thus, the CALIOP values close to the tropopause in Fig. 1 during 2006 and 2007 won’t be interpreted.

### Volcanism

The three major eruptions of Kasatochi (7^th^ August 2008), Sarychev Peak (12^th^ June 2009) and Nabro (12^th^ June 2011) in Table 2 are seen in Fig. 1 to increase the aerosol backscattering according to both CALIOP and IAGOS-CARIBIC for several months after the initial eruptions. Since it takes some weeks for the aerosol to grow and spread, the maximum effect on the aerosol backscattering is seen in the months following an eruption. The elevated values in June and July 2011, from both CALIOP and IAGOS-CARIBIC, were caused by the eruption of Grimsvötn (21^th^ May 2011). From Fig. 1 it was determined which months following Kasatochi, Sarychev, Grimsvötn and Nabro was influenced by volcanism and this information will be used to label months for subsequent analysis.

**Table 2:** Volcanic eruptions between 2006 and 2015 which entered the NH stratosphere.

Eruption Date	Volcano	Volcanic Explosivity Index^88^	Tg SO_2_	Latitude, longitude
20 May 2006	Soufrière Hills	3	0.1^89^; 0.2^90^	16.7°N, 62.2°W
7 October 2006	Rabaul	4	0.23^91^; 0.13^92^	4.3°S, 152°E
12 July 2008	Okmok	4	0.12^93^	55.3°N, 168.2°W
7 August 2008	Kasatochi	4	2^94^; 1.7^93^	52.2°N, 176°W
20 March 2009	Redoubt	3	0.23^95^	60.5°N, 153°W
12 June 2009	Sarychev	4	1.2^96^	48.1°N, 153°E
21 May 2011	Grimsvötn	4	0.4^97^	54.4°N, 17.3°E
12 June 2011	Nabro	4	1.5^97^	13.4°N, 41.7°E
14 February 2014	Kelut	4	0.17^98^	7.9°S, 112.3°E

Of the minor eruptions, here defined as SO_2_ load smaller than 0.25 Tg, it was Redoubt (starting 20^th^ March 2009) that increased the aerosol backscattering the most. In the two months following the Redoubt eruptions, both CALIOP and IAGOS-CARIBIC show small increases in the aerosol backscattering. The tropical volcano Kelut erupted on 14^th^ February 2014, but no signal different to what is usually seen during spring was seen in the following three months since it did not subside to 12 km in this study’s timeframe^11^. The Kelut aerosol and precursor gases were injected deeply into the stratosphere and most of its effluents rose in the tropical pipe^11^.

**Figure 2:**
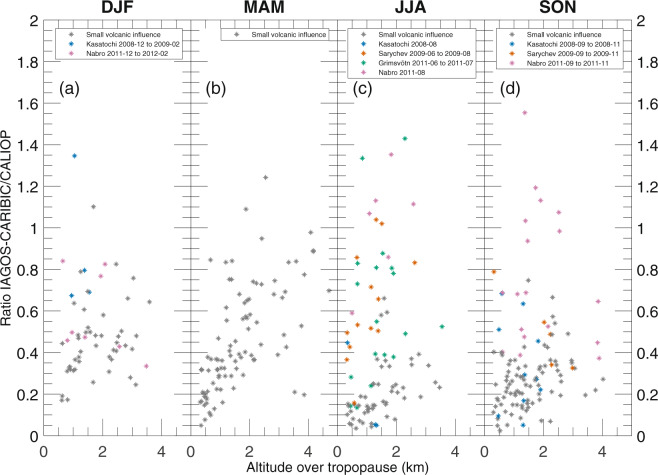
Ratios between the IAGOS-CARIBIC and monthly mean CALIOP aerosol backscattering from Fig. 1 for all years combined plotted against altitude above the tropopause. Data points strongly affected by volcanism are coloured according to specific eruption, whereas months when the volcanic influence is small are marked grey.

The ratios between the IAGOS-CARIBIC and CALIOP aerosol backscattering at the IAGOS-CARIBIC altitudes above the tropopause are shown in Fig. 2, where the measurements that were most influenced by volcanic eruptions are highlighted by colour, and data from all years have been plotted in the same subfigures. Months with data less affected by volcanism are marked grey and will be discussed below. Volcanic eruptions induce a patchiness in the aerosol concentration that affects point measurements, like those of IAGOS-CARIBIC. The CALIOP measurements, as presented here, are less affected, because monthly means over the entire latitude interval (30–70°N) are used (Fig. 2). The ratio in aerosol backscattering tend to be higher when the volcanic influence increases, this will be further investigated in the Discussion section.

### Seasonal variation in scattering according to aerosol samples and lidar

**Figure 3:**
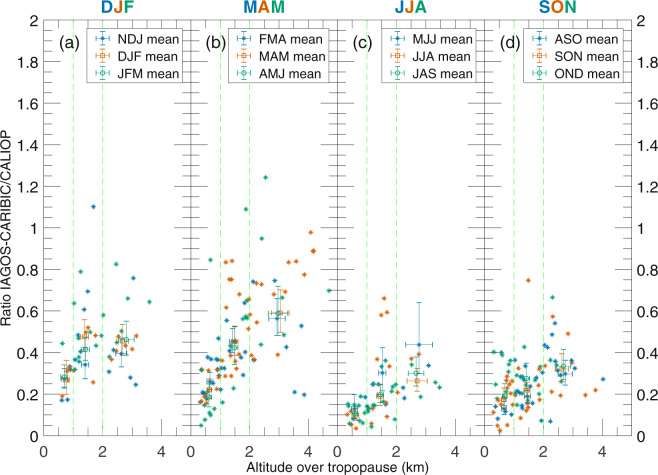
Ratios between the IAGOS-CARIBIC and monthly mean CALIOP aerosol scattering from Fig. 1 for all years combined related to the altitude above the tropopause. Data from months more affected by volcanism have been excluded (compare Fig. 2). Mean values together with double-sided 95% confidence intervals, calculated on the logarithm of ratios using Student’s t-distribution and then transformed back, for the mean ratio and altitude have been calculated for each month in three altitude intervals, 0.3–1 km, 1–2 km and above 2 km above the tropopause.

**Figure 4:**
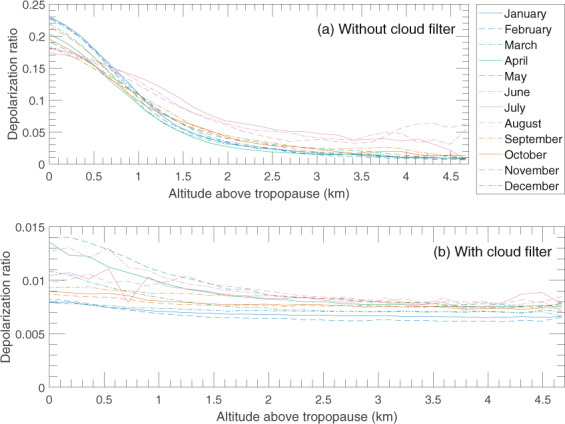
Monthly means of depolarisation ratio for the years 2006–2014 when the influence from volcanism was small both without the cloud filter (**a**) and with the cloud filter (**b**). The average perpendicular and total backscattering for each month was calculated over the entire time span before the depolarization ratio for this plot was calculated. Note the different scales on the ordinate of the two subfigures.

We now continue with the ratio between IAGOS-CARIBIC and CALIOP aerosol backscattering, but without data from months strongly affected by volcanism. Figure 3 contains the ratios that were present as grey marks in Fig. 2. Generally, the ratio increases with height above the tropopause. The low ratios close to the tropopause relate to the monthly average depolarization ratios in Fig. 4, which indicate the increase in tropospheric influence closer to the tropopause both with and without the cloud filter. Note the different scales on the ordinate for the two subfigures in Fig. 4. The cloud filter was applied to the CALIOP data in all other analysis in this study.

Figure 3 also indicates a seasonal variation in the ratio between IAGOS-CARIBIC and CALIOP aerosol backscattering. To study this, we divided the samples into the altitude intervals 0.3–1, 1–2 and above 2 km above the tropopause and calculated geometrical averages of the ratios and arithmetical averages of the altitude above tropopause. The average altitude within the altitude intervals varies only slightly over the season in Fig. 3. This means that it is possible to compare average ratios for different months since they are still located at approximately the same height above the tropopause. We used three-month moving geometric averages since the number of IAGOS-CARIBIC samples is small in the upper two altitude intervals for some months.

**Figure 5:**
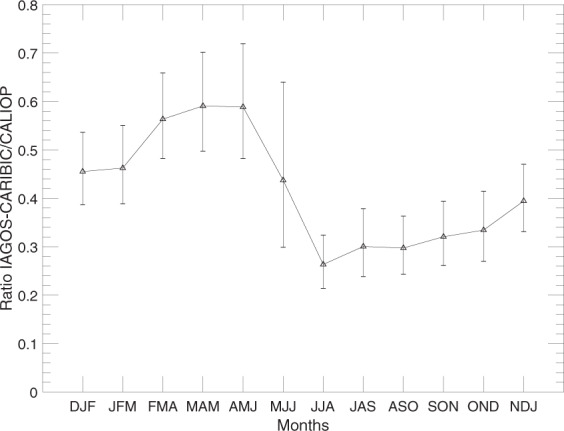
Geometrical averages and double-sided 95% confidence interval for the average ratio of aerosol scattering of IAGOS-CARIBIC over CALIOP at altitudes 2 km above the tropopause from Fig. 3. The confidence intervals were computed based on logarithms of the ratios and transformed back. They are symmetrical in a log-scale, and therefore somewhat longer upwards in a linear scale.

**Figure 6:**
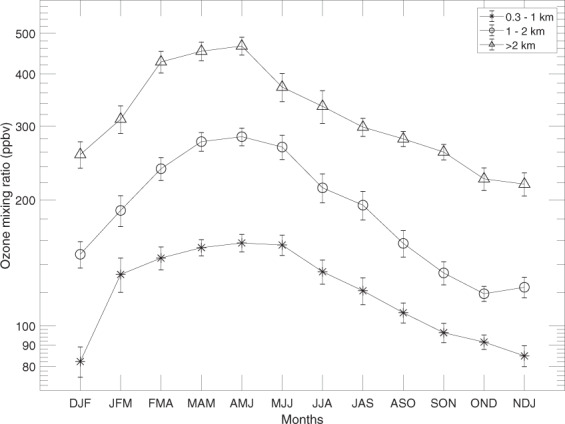
Geometrical average ozone mixing ratios measured with IAGOS-CARIBIC. The error bars indicate the standard error.

From Fig. 3 it is clear that the agreement between the two measurement methods is poor close to the tropopause. Therefore, the seasonal variation in aerosol backscattering ratios (data from Fig. 3) will only be investigated in the altitude interval 2 km above the tropopause in Fig. 5. The particles in the highest altitude interval are, according to air motion patterns as well as the depolarisation ratio of the LMS particles (Fig. 4), most influenced by the stratosphere. To further underline the stratospheric influence, the tracer of stratospheric air, O_3_, measured by IAGOS-CARIBIC, is shown in Fig. 6. As can be seen in Fig. 5, the best agreement between the two methods are obtained in the winter and spring. This will, together with the poor agreement close to the tropopause, be further discussed in the Discussion section.

### Sensitivity analysis

The comparison between the aircraft- and satellite-based aerosol measurements is affected by uncertainties in concentrations, composition, physio-chemical and optical properties of the aerosol. Here we will investigate the sensitivity to changes in these parameters for the backscatter ratio in the layer deepest into the LMS (2 km or more above the tropopause), starting with the particle size distribution. The uncertainty in the IAGOS-CARIBIC particle diameter measurements is estimated to be 15% due to uncertainties in the refractive index^39^. The standard deviation in the volume geometrical mean diameter over the year studied (Methods section) was 6.7%, combining to ±16% error interval in particle size. Changing the size accordingly affected the backscattering by −4.4% and +7.3%, see Table 3. In this size range more backscattering is produced by fewer but larger particles than more but smaller particles for a given aerosol mass concentration. The geometrical standard deviation of the size distributions varied by ±3.4% over the year investigated, leading to changes in the backscattering by −1.9% and +1.6%.

**Table 3:** Sensitivity study of backscattering computed from IAGOS-CARIBIC aerosol samples.

Parameter	Base case	Case 1	Case 2	Δ_backscat_1	Δ_backscat_2
*D*_*g,v*_ (nm)^a^	321	270	372	−4.4%	7.3%
Geometric standard deviation	1.52	1.47	1.57	−1.9%	1.6%
Black carbon (ng/kg)	1	2	0	−5.6%	6.3%
Organic/Carbon mass	1.25	1.05	1.45	−5.9%	5.9%
Organic density (kg/m^3^)	1200	1400	1000	−5.3%	7.4%
Organic refractive index	1.55–0i	1.5–0i	1.6–0i	−6.1%	6.5%
Error in RH (fraction base rel. case)	1	0.919	1.081	−0.7%	0.7%
Sampling and analytical error	—	—	—	−12%	12%
Total error	—	—	—	−17%	19%

^a^Geometric volume mean diameter.

The BC concentration, which was not measured in the time period of this study, constitute a major uncertainty. Relying on literature data^53–56^ the base case was set to 1 ng kg^−1^ with a wide (±100%) test range, leading to −5.6% with doubled BC, and without BC +6.3% change in the backscattering. The organic aerosol mass concentration was estimated to 1.25 times the carbon concentration based on stoichiometric relations (Methods section) with variations in the factor by ±0.2, leading to ±5.9% change in the backscattering. The density of the organic fraction affects the estimated particle volume. Here it was tested in the range 1200 ± 200 kg/m^3^ ^50–52^, affecting the backscattering by −5.3% for 1400 kg m^−3^ and +7.4% for 1000 kg m^−3^.

Several measurements from various locations report on a wavelength-dependent absorption of organic aerosol, being strongest in the UV and shorter visible wavelengths^61^. The effect at the CALIOP wavelength (532 nm) is usually smaller, and given that very little is known about the composition and optical properties of carbonaceous fraction of the stratospheric aerosol the possible absorption is not investigated. Instead, only scattering of the organic aerosol with a real refractive index of 1.55 ± 0.05^61–63^ is investigated, inducing −6.1% and +6.5% change in the backscattering. It should be pointed out that density and refractive index usually are correlated^73^, thus leading to an overestimation of the combined error here where they are treated as independent.

The uncertainties in the relative humidity computations were estimated based on measurement uncertainties in the gas-phase water concentration measurements (Methods section), uncertainties in atmospheric pressure and temperature measurements^74^, and uncertainties in thermo-dynamical data needed to obtain saturation vapour pressure, H_2_SO_4_/H_2_O mixture, and its density. To account for these uncertainties, the error in the gas-phase water were doubled. As pointed out before, the properties of the organic fraction of the stratospheric aerosol is not well known. Here we assume that it does not affect the water uptake. The uncertainties in the hygroscopic growth is estimated to induce ±0.7% uncertainty in the backscattering.

The overall uncertainties from these seven tests are estimated to −12% to +14%, handling the different tests as independent. They should be combined with the uncertainties in sampling and analysis of 12%, leading to an overall uncertainty in the range −17% to +19%. In this sensitivity analysis we have varied the parameters in reasonable intervals. Still we find that the ratio between the backscattering obtained from the IAGOS-CARIBIC samples to that of CALIOP deviates significantly from unity, and vary with distance from the tropopause and with season. A discussion of these deviations will follow next.

## Discussion

During winter and spring we can expect to see more stratospheric air delivered by the stronger Brewer-Dobson circulation^75^ and less tropospheric air due to strong cross-tropopause blocking around the sub-tropical jet^76^. We see higher IAGOS-CARIBIC over CALIOP backscatter ratios, less depolarisation and higher O_3_ concentrations above 2 km of the tropopause (Figs 3–6), all of which are indicative of these effects. Bönisch, *et al*.^77^ also found that the tropospheric influence in the LMS is smallest during spring. Furthermore, ozone levels show clear peaks in spring and lower respective values in autumn (Fig. 6) illustrating the extent of stratospheric influence in the LMS.

Conversely, during summer and autumn we can expect to see less stratospheric air delivered by a weaker Brewer-Dobson circulation and more tropospheric air because of the weakening of the sub-tropical jet block. Tropospheric air is the main component of the LMS during summer and fall^77^. The weaker blocking at the sub-tropical jet in the summer combined with strong convective activity contribute to the formation of the Asian Tropopause Aerosol Layer (ATAL), extending across the LMS^78^. The lower backscatter ratios between the two methods during summer coincides with more depolarisation in the CALIOP signal (Fig. 4) and lower O_3_ concentrations connected with a strong tropospheric influence.

**Figure 7:**
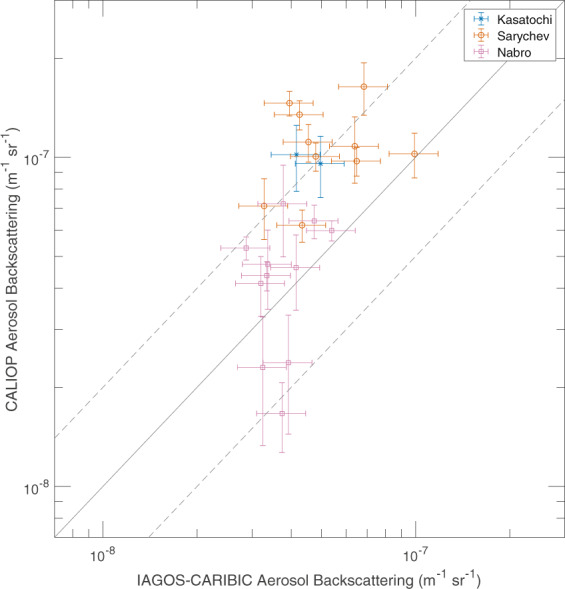
Aerosol backscattering from CALIOP swaths taken near individual IAGOS-CARIBIC samples. The error bars on the ordinate are 95% confidence intervals for the CALIOP averages and the error bars on the abscissa are the uncertainties from the sensitivity analysis of IAGOS CARIBIC.

The IAGOS-CARIBIC samples strongly affected by volcanism are characterized by patchiness in the aerosol concentration. Therefore, we also used an additional, alternative approach for analysing these data. Instead of comparing monthly CALIOP averages over large areas to several IAGOS-CARIBIC samples, we compare individual IAGOS-CARIBIC samples to nearby CALIOP swaths (Fig. 7). The CALIOP swaths used for each sample was in a latitude-longitude box, measuring 15° in latitude and 50° in longitude, around an aerosol sample with the additional conditions that swaths have to be taken within one day of the IAGOS-CARIBIC sampling and that at least 8° of latitude in the box has to be covered by CALIOP swaths in order for a comparison to be made. Figure 7 shows that the agreement between the two methods tend to be better for the Nabro eruption in 2011 than for the 2009 Sarychev eruption. Overall, the observation systems are within a factor of two for 65% of the samples and the average IAGOS-CARIBIC over CALIOP backscattering ratio is 0.75. The average ratio between the calculated backscattering based on IAGOS-CARIBIC measurements to that measured by CALIOP is closer to unity when the volcanic influence is strong compared to the samples with less volcanic influence. This is in line with the better agreement for sulphur-rich samples during periods of less volcanic influence.

We have attempted to calculate backscattering for one wavelength from *in situ* measured and assumed aerosol particle properties. However, calculation of backscattering requires complete information on the aerosol particles. From Fig. 5 it is clear that our calculation results based on IAGOS-CARIBIC data do not capture all of the backscattering that CALIOP measures. The calculated aerosol backscattering from IAGOS-CARIBIC can be as little as 10% of what CALIOP measures and the difference between the results from the two systems can’t be fully explained by the uncertainties discussed above. One reason for this could be that volatile aerosol components, such as semi-volatile organic compounds, are evaporated in the approximately 30 °C sampling line in IAGOS-CARIBIC and thus not accounted for. In the first round of the Balloon Measurements of the Asian Tropopause Aerosol Layer (BATAL), a large fraction of nitrate at the tropical tropopause was found^79^. Höpfner, *et al*.^80^ also found large fractions of nitrate in the upper troposphere. In our measurements, this nitrate could, together with semi-volatile organics, have been vaporised.

Another explanation for the disagreement could be the components that are not included in our stratospheric model. The vertical gradients for the CALIOP backscattering vary, with both positive and negative ones, because the lowest part of the LMS contains strong gradients of species having different concentrations in the troposphere and stratosphere. A vertical gradient for depolarization ratio after the cloud filter can also be seen (Fig. 4). Proestakis, *et al*.^81^ saw mineral dust in Asia reaching altitudes up to 10 km, which is inside our studied altitude interval, and Trickl, *et al*.^82^ saw that Asian dust storms could be at altitudes between 7 and 13 km. Although dust is depolarising it is possible that some could have been let through the CALIOP cloud filter. Murphy, *et al*.^83^ showed that there are fractions of non-volatile material in the tropospheric aerosol at all altitudes and that nitrate is usually found in silicon containing particles. IAGOS-CARIBIC detect elements characteristic of mineral dust in aerosol samples taken in the LMS^84^. However, these results cannot be used quantitatively due to sampling problems^42^ and the particle size range not being optimized for crustal particles^41^. Therefore, mineral dust is not covered by our aerosol model, which could be a partial explanation for the low IAGOS-CARIBIC over CALIOP ratios. Wang, *et al*.^85^ summarized observations of how convective storms can inject water vapour and ice particles into the stratosphere. It is possible that some clouds could have passed our cloud filter, especially since Spang, *et al*.^86^, using data from Pan and Munchak^87^, showed that CALIOP can detect fewer thin cirrus clouds in the stratosphere than the Cryogenic Infrared Spectrometers and Telescopes for the Atmosphere (CRISTA).

In conclusion, it is difficult to deduce microphysical aerosol properties with high accuracy from lidar measurements. The aerosol model used here is based on measured size distributions, but does not cover all components of the tropospheric aerosol. Heating of the inlet and sample handling at room temperature, which is significantly higher than the temperature in the LMS, leads to losses of semi-volatile aerosol components^42^. The agreement between the two methods compared vary from poor to reasonably good, where the latter is valid for aerosol containing a strong sulphurous component.

## Conclusions

The primary goal of this study is to evaluate the compatibility of two long-term stratospheric aerosol data sets: the lidar measurements of the CALIOP sensor aboard the CALIPSO satellite and elemental concentrations from aerosol samples together with ozone, H_2_O and particle size measurements collected during intercontinental flights from the IAGOS-CARIBIC observatory. The study covers the time period (2006–2014) and altitude range (9.5–12 km) in the northern hemisphere (30–70°N) where data are available from both methods. The altitude range implies that the study pertains to the lowermost part of the stratosphere (LMS). Based on measured concentration of particulate sulphur and carbon, water in the gas phase inducing hygroscopic growth and particle size distributions from IAGOS-CARIBIC, and literature data on the black carbon concentration, the backscattering was modelled for the comparison, assuming particles consisting of sulphuric acid/water, organics and black carbon.

The LMS was investigated in three layers: 0.3–1, 1–2 and more than 2 km above the dynamical tropopause, here chosen at 1.5 PVU. The relative humidity on average was 27% and 4% in the lower and higher layers, corresponding to 47% and 65% of sulphuric acid in the sulphurous part of the particles, respectively. The size distributions were measured at room temperature. This temperature increase reduced the volume of the sulphuric acid/water by on average a factor of 2.3 and 1.6 for the lower and upper layer, implying that the atmospheric particle size is larger than the measured size. The refractive index (RI) was computed based on a volume mixing rule. The sulphuric acid/water RI are obtained from computations relating to composition, whereas organics and black carbon refractive indices are based on literature data. A sensitivity study of the backscattering obtained from the modelling of the IAGOS-CARIBIC data is estimated to ±20% in standard deviation.

Forming the ratio of IAGOS-CARIBIC over CALIOP backscattering, we found values as low as 0.1 close to the tropopause. The ratio between the two methods increases with height above the tropopause as the influence from tropospheric aerosol decreases. In the layer from 2 km above the tropopause the monthly average ratio reaches at most 0.6. The highest ratio between the two methods is obtained for winter and spring measurements coinciding with strong transport down from the stratosphere and thus a larger stratospheric fraction in the LMS air. Conversely, there are lower ratios between the two data sets during summer and autumn. During these seasons there is less transport down from the stratosphere and more transport into the LMS from the subtropical troposphere (above the 350 K isentrope), thus also making the deeper LMS more tropospheric in terms of origin and composition. The degree of agreement between the two methods is low when the tropospheric influence is strong, whereas the highest ratios appear when the stratospheric influence is strong. These observations were made for periods of rather weak volcanic influence. During the studied period, the stratosphere, including the LMS, was affected by medium-sized volcanic eruptions, notably Kasatochi (2008), Sarychev (2009) and Nabro (2011). The backscattering ratio between the two methods were higher when the LMS was affected by these eruptions, on average 0.75, compared with stratospheric conditions close to background.

Discrepancies between the methods can be caused by faint cloud residues not completely removed by the cloud mask used for CALIOP and a presence of aerosol components, such as crustal particles or nitrate, not accounted for in the backscattering modelled for the IAGOS-CARIBIC aerosol. Such a presence is indicated by a strong increase in the CALIOP depolarization ratio close to the tropopause. Additional explanations could be evaporative losses during particle sizing and chemical analyses that took place at strongly elevated temperature (room temperature) compared with atmospheric conditions.

To our knowledge this is the first time *in-situ* stratospheric aerosol measurements have been compared to space-based lidar measurements of the CALIOP instrument. This study indicates that it is possible to link the near global satellite measurements of CALIOP with the aircraft measurements of IAGOS-CARIBIC for stratospheric aerosol, whereas a substantial disagreement is found when the influence from tropospheric air is strong.

## Data Availability

Data for producing the figures in this article are available upon reasonable request from the corresponding author.
